# Prevalence of physical abuse of children in their homes in Ilorin Nigeria

**DOI:** 10.1192/j.eurpsy.2021.1886

**Published:** 2021-08-13

**Authors:** A. Oladosu, O. Abiodun, M. Tunde-Ayinmode

**Affiliations:** 1 Johnson Community Hospital, Lincolnshire Partnership Foundation Trust, Lincolnshire, United Kingdom; 2 Behavioural Sciences, University of Ilorin Teaching Hospital, Ilorin, Nigeria

**Keywords:** child, physical abuse, home

## Abstract

**Introduction:**

Child abuse has deleterious consequences on its victims. Its occurrence is poorly documented in Nigeria.

**Objectives:**

To determine prevalence and pattern of physical abuse at home among children in Ilorin Nigeria.

**Methods:**

Cross sectional survey of secondary school students aged 11-18 years in Ilorin Nigeria using multistage random sampling technique with proportional allocation was done. Respondents completed the ICAST-CH questionnaire which covers child abuse in its several forms. Prevalence of child abuse was computed.

**Results:**

Table1: Pattern of physical abuse at home in the last 12 months

**Table:** 

Form of abuse	Frequency	Percentage
Physical Abuse* (n=1554) Hold heavy load as punishment/positional fixity)	1492	96.0
Hit with object	1473	94.8
Hit, beat, spanked with hand	1203	77.4
Pushed, grabbed, kicked	850	54.7
Pulled hair, pinched, twisted ear	631	40.6
Locked in small place	182	11.7
Burned or scalded	85	5.5
Tried to choke, smother, or drown	81	5.2
Threatened with knife or gun	30	1.9

**Conclusions:**

Conclusion Physical abuse of children is extremely common in Ilorin Nigeria. There are no specific demographic determinants of occurrence; hence every growing child is at risk. The prevailing cultural norms and state laws appear to be chief drivers of this phenomenon. The current findings expand the available pool of knowledge about CPA in Nigeria and calls for more research. It also supports existing calls for the abolition of corporal punishment of children.

**Disclosure:**

No significant relationships.

